# Metabolome analysis reveals a role for glyceraldehyde 3-phosphate dehydrogenase in the inhibition of *C. thermocellum* by ethanol

**DOI:** 10.1186/s13068-017-0961-3

**Published:** 2017-11-30

**Authors:** Liang Tian, Skyler J. Perot, David Stevenson, Tyler Jacobson, Anthony A. Lanahan, Daniel Amador-Noguez, Daniel G. Olson, Lee R. Lynd

**Affiliations:** 1 Thayer School of Engineering, Dartmouth College, 14 Engineering Drive, Hanover, NH 03755 USA; 2 Dartmouth College, Hanover, NH 03755 USA; 3 Bioenergy Science Center, Oak Ridge National Laboratory, Oak Ridge, TN 37831 USA; 4 University of Wisconsin-Madison, Madison, WI 53706 USA

**Keywords:** Consolidated bioprocessing, *Clostridium thermocellum*, Ethanol tolerance, Metabolomic analysis

## Abstract

**Background:**

*Clostridium thermocellum* is a promising microorganism for conversion of cellulosic biomass to biofuel, without added enzymes; however, the low ethanol titer produced by strains developed thus far is an obstacle to industrial application.

**Results:**

Here, we analyzed changes in the relative concentration of intracellular metabolites in response to gradual addition of ethanol to growing cultures. For *C. thermocellum*, we observed that ethanol tolerance, in experiments with gradual ethanol addition, was twofold higher than previously observed in response to a stepwise increase in the ethanol concentration, and appears to be due to a mechanism other than mutation. As ethanol concentrations increased, we found accumulation of metabolites upstream of the glyceraldehyde 3-phosphate dehydrogenase (GAPDH) reaction and depletion of metabolites downstream of that reaction. This pattern was not observed in the more ethanol-tolerant organism *Thermoanaerobacterium saccharolyticum*. We hypothesize that the Gapdh enzyme may have different properties in the two organisms. Our hypothesis is supported by enzyme assays showing greater sensitivity of the *C. thermocellum* enzyme to high levels of NADH, and by the increase in ethanol tolerance and production when the *T. saccharolyticum gapdh* was expressed in *C. thermocellum*.

**Conclusions:**

We have demonstrated that a metabolic bottleneck occurs at the GAPDH reaction when the growth of *C. thermocellum* is inhibited by high levels of ethanol. We then showed that this bottleneck could be relieved by expression of the *gapdh* gene from *T. saccharolyticum*. This enzyme is a promising target for future metabolic engineering work.

**Electronic supplementary material:**

The online version of this article (10.1186/s13068-017-0961-3) contains supplementary material, which is available to authorized users.

## Background

Plant biomass is of interest as a feedstock for sustainable production of liquid fuels and organic chemicals [1]. Consolidated bioprocessing (CBP), in which biomass solubilization and fermentation are accomplished in one step, without added enzymes, is a promising processing configuration for low-cost biological conversion of plant biomass [2–5]. As a candidate organism for CBP, *Clostridium thermocellum* is among the most effective microorganisms, described to date, for lignocellulose deconstruction [6, 7]. Different approaches have been applied to engineer *C. thermocellum* to produce ethanol at high yield and titer, including eliminating native by-products, overexpressing native genes, introducing heterologous genes, and adaptive evolution [8–13]. However, the highest reported ethanol titer produced by this microbe in pure culture is 27 g/L, which is below the titer of 40 g/L widely assumed to be necessary for commercial application [14]. Substantially higher titers, e.g., 70 g/L, have been produced by engineered strains of *Thermoanaerobacterium saccharolyticum*, a hemicellulose-fermenting thermophilic anaerobe [15].

Ethanol inhibition has been extensively studied in bacteria, and several mechanisms have been proposed, including membrane fluidization, disruption of proton motive force and ATP generation, disruption of nicotinamide cofactor ratios, and inhibition of key metabolic enzymes [16, 17]. Wild-type *C. thermocellum* is unable to initiate growth when inoculated into medium containing ethanol at concentrations of 20 g/L or higher [10, 18, 19]. Strains adapted for improved tolerance by serial transfer over a period of several weeks have been shown to initiate growth in the presence of 50–55 g/L ethanol [18–22]. In *C. thermocellum*, the mechanism of tolerance has been attributed to both changes in membrane properties [19, 20, 23, 24], and metabolic enzymes, in particular, the bifunctional acetaldehyde-CoA/alcohol dehydrogenase gene (*adhE*) [18, 21]. In a similar organism, *Thermoanaerobacter thermohydrosulfuricus,* Lovitt et al. found that selection for ethanol tolerance resulted in several metabolic changes, including the elimination of NADH-linked alcohol dehydrogenase (ADH-NADH) activity, elimination of Ferredoxin-NAD^+^ activity, and a change in the properties of the Gapdh enzyme that made it less sensitive to inhibition by NADH [25]. The genetic basis of the elimination of ADH-NADH activity was not determined; however, it is reasonable to suspect that this may have been due to a mutation in the *adhE* gene.

Although ethanol tolerance has often been studied as a proxy for ethanol production, many studies have found that increases in ethanol tolerance have no effect on ethanol production [26–28], including studies of *C. thermocellum* [29]. Furthermore, in cases where ethanol tolerance has been improved by selection, many of the improvements appear to be due to idiosyncratic mutations whose effects are not generalizable to other strain backgrounds or growth conditions [17, 26].

Thus, we have focused our inquiry on facets of ethanol tolerance that are relevant to high titer production, such as metabolism. Recent advances in analytical chemistry have allowed for measurement of many of the intracellular metabolites involved in glycolysis and fermentation (i.e., metabolomics) [30]. Metabolomic analysis has been used to investigate the short-term effect of ethanol inhibition on *C. thermocellum* by Yang et al. [31]. They found that a pulse of added ethanol (“ethanol shock”) of about 4 g/L was sufficient to temporarily halt growth, and found intracellular accumulation of fructose-6-phosphate and glucose-6-phosphate. Since they only measured two intracellular metabolites related to glycolysis and fermentation, however, they were unable to precisely determine the location of the metabolic disruption. Furthermore, since *C. thermocellum* is known to be able to tolerate much more than 4 g/L ethanol, it suggests that at least some of the effects observed by Yang et al. were due to the sudden addition of ethanol rather than its absolute titer.

Factors associated with the cessation of fermentation in *C. thermocellum* include accumulation of ethanol [10] and other fermentation products [32] as well as nutrient limitation [33]. Accumulation of salt added to neutralize acid production was found to limit fermentation by *Thermoanaerobacterium thermosaccharolyticum* at high-feed xylose concentrations in continuous culture [34]. Fermentation in *C. thermocellum* is repeatedly observed to stop at ethanol concentrations well below those that can be tolerated by adapted strains. This phenomenon, referred to as a “titer gap” [3], remains to be explained. In this work, we applied the tools of metabolomics and genetic engineering to pursue the question: “what limits ethanol production at high titer?”

## Methods

### Bacterial strains, media, and growth conditions

Strains used in this study are listed in Additional file 1: Table S1. Plasmids and primers are listed in Additional file 2: Table S2 and Additional file 3: Table S3. All chemicals were reagent grade or better and obtained from Sigma-Aldrich (St. Louis, MO, USA) or Fisher Scientific (Pittsburgh, PA, USA) unless indicated otherwise. CTFUD-rich medium at pH 7.0 and pH 6.0 was used for *C. thermocellum* and *T. saccharolyticum* strain maintenance, respectively [35]. For metabolomic analysis, *C. thermocellum* was grown LC medium [36] and *T. saccharolyticum* was grown in modified MTC-6 medium [9, 37]. The growth temperature was 55 °C for both strains. Bioreactor fermentations to measure ethanol inhibition were carried out in 250-mL bioreactors (NDS, Vineland, NJ, USA).

Avicel fermentations to measure ethanol production were carried out in 1.5-L (1-L working volume) Sartorius Biostat A-plus Sartorius Stedim (Sartorius Stedim, Bohemia, NY, USA) bioreactors in modified MTC-5 medium without MOPS buffer and with 2 g/L urea as the nitrogen source, with the temperature maintained at 55 °C and stirred at 150 rpm. The pH was controlled at 6.5 with a Mettler-Toledo pH probe (Columbus, OH, USA) by the addition of 8 N KOH [10].

### Ethanol tolerance assay

Each strain was cultured in a single bioreactor until the OD_600_ reached 0.1–0.2. Then the culture was split and transferred into two bioreactors (we refer to this as time T0). At this time, a feed of pure, deoxygenated ethanol was pumped into one of the bioreactor at the rate of 5 g/L/h, meanwhile, deoxygenated water was pumped to the other bioreactor as control. A Cole-Parmer peristaltic pump was used for pumping (Vernon Hills, IL, USA). Both the ethanol and water were kept in serum bottles and purged with pure N_2_ gas before using. Since ethanol and water have different densities, the volumetric flow rate of each pump had to be calibrated independently to assure identical mass flow rates.

Intracellular metabolite samples were collected by the filter technique described by Olson et al. [38]. The extraction solution was a 2:2:1 ratio of acetonitrile:methanol:water. A volume of 2–10 mL of culture was transferred to a 0.45-µm nylon membrane filter (EMD Millipore, Billerica, MA, USA) and vacuum-filtered. After excess medium had been removed, the filter was placed in a plastic petri dish with 1.6 mL cold extraction solution to quench metabolism and extract the metabolites. The extraction solution was kept cold by being placed in contact with a 4-inch-thick aluminum block prechilled to − 80 °C. Sampling of the reactor, filtration, and quenching were all performed in an anaerobic chamber (COY, Grass Lake, MI, USA) with an atmosphere of 85% N_2_, 10% CO_2_, and 5% H_2_ to avoid oxygen exposure during metabolite sampling.

The petri dishes with extraction solution, filter, and cells were placed at − 80 °C for 1–24 h to allow a pause in the protocol. They were then thawed and the cells were washed off of the filter by pipetting, using gentle scraping as needed. The cells and extraction solution were transferred to 1.5-mL microcentrifuge tubes and centrifuged at 15,000×*g* for 1 min. The supernatant was collected and analyzed by LC–MS to identify and quantify metabolites.

### Metabolomics analysis

Samples were analyzed using an LC–MS/MS system consisting of a Dionex Ultimate 3000 UHPLC coupled by electrospray ionization (ESI; negative mode) to a hybrid quadrupole—high-resolution mass spectrometer (Q Exactive orbitrap, Thermo Scientific) for detection of targeted compounds based on their accurate masses and retention times (matched to purified standards). Liquid chromatography (LC) separation was achieved using an ACQUITY UPLC^®^ BEH C18 (2.1 × 100 mm column, 1.7-μm particle size) and a flow rate of 0.2 mL/min. Solvent A was 97:3 water:methanol with 10 mM tributylamine (TBA) and approximately 9.8 mM acetic acid, pH ~ 8.2; solvent B was 100% methanol. Total run time was 24.5 min with the following gradient: 0 min, 5% B; 2.5 min, ramp from 5% B to 95% B over 14.5 min; hold at 95% B for 2.5 min; return to 5% B over 0.5 min; hold at 5% B for 5 min. All samples were injected twice (analytical replicates). MS scans consisted of full negative mode MS scanning for *m/z* between 70 and 1000 from time 0 to 18.5 min. Metabolite peaks were identified using Metabolomics Analysis and Visualization Engine (MAVEN) [39, 40]. Response factors from the standards were used to calculate absolute values of the nicotinamide cofactors and adenosine phosphate cofactors.

### qPCR for cell number measurement

Metabolite concentrations were normalized to cell number. Cell number was determined by qPCR as follows: after taking an initial sample of culture for metabolite measurements, a second sample of culture was taken for cell number measurements. The cells were transferred to a 0.45-µm nylon membrane filter and vacuum-filtered, then transferred to a plastic petri dish with 0.5 mL TE buffer. The cells were washed off of the filter by pipetting, then transferred to a 1.5-mL microcentrifuge tube and washed twice with TE buffer. The washed cells were suspended in 0.5 mL TE buffer and quantified by qPCR. Serial dilution of the samples was performed to ensure the qPCR signal was within the range of the standard curve.

A synthesized fragment of double-stranded DNA (gBlock^®^, IDT, Coralville, Iowa, USA) was used as standard to quantify DNA levels. The gBlock used for this assay contained part of the *recA* gene sequences from both *C. thermocellum* and *T. saccharolyticum*. The amplicon for each gene was around 120 and 5 bp was added on both sides of each amplicon to avoid secondary structure. The gBlock was first diluted to a concentration of 10 ng/µL (12 nM) and five tenfold dilutions were prepared to make the standard curve.

SsoFast™ EvaGreen^®^ Supermix (Bio-Rad, Hercules, CA, USA) was used for the qPCR reaction and the assay was run in triplicate in a 10 µL reaction volume. The number of cells was determined by comparison of copy number of the *recA* gene with the standard curve, using the assumption that each cell had one copy of its chromosome.

### Analytical methods

Acetate, formate, ethanol, glucose, and residual cellobiose were determined by high-pressure liquid chromatography (HPLC, Waters, Milford, MA) with refractive index detection using an Aminex HPX-87H column (Bio-Rad, Hercules, CA) with a 2.5 mM sulfuric acid solution mobile phase. Pellet nitrogen was determined using a Shimadzu TOCV-CPH total organic carbon analyzer with added total nitrogen unit (Shimadzu Scientific Instruments, Columbia, MD, USA), calibrated using an acidified glycine standard [41].

### Heterologous expression of protein in *E. coli*

The *gapdh* genes from *C. thermocellum* and *T. saccharolyticum* were amplified by PCR with Q5 DNA polymerase (New England Biolabs, Ipswich, MA, USA). The primers used for each gene are listed in Additional file 3: Table S3. Target genes were inserted into plasmid pD861-NH (DNA2.0 Inc., Menlo Park, CA, USA) and tagged with a 5× His6 cassette. The vector was transformed into *E. coli* BL21(DE3). Cells were grown aerobically in TB medium (Sigma T0918, St. Louis, MO, USA) at 37 °C with a stirring speed of 225 rpm [9]. When the OD_600_ reached 0.6, 4 mM rhamnose was added to induce the expression of the target gene. The cells were then grown aerobically for 4 h before harvesting by centrifugation. The cell pellets were washed with 50 mM Tris–HCl with 0.5 mM DTT (pH 7.5) and stored at − 80 °C.

The cell pellet was resuspended in lysis buffer (50 mM sodium phosphate pH 7.5, 500 mM NaCl, 20 mM imidazole, 1× BugBuster reagent (EMD Millipore, Darmstadt, Germany), and 0.2 mM dithiothreitol [DTT]). The cells were lysed with Ready-Lyse lysozyme (Epicentre, Madison, WI, USA), and DNase I (New England Biolabs, Ipswich, MA, USA) was added to reduce the viscosity. After incubation for 30 min. at room temperature, the resulting solution was centrifuged at 10,000×*g* for 5 min. The supernatant was used as cell extract for enzyme purification. Native *E. coli* proteins were denatured by incubation at 55 °C for 30 min. The denatured proteins were then removed by centrifugation at 10,000×*g* for 5 min. His tag affinity spin columns (His SpinTrap; GE Healthcare Bio-Sciences, Pittsburgh, PA, USA) were used to purify the protein. The column was first equilibrated with binding buffer (50 mM sodium phosphate, 500 mM NaCl, 20 mM imidazole, pH 7.5). Cell extracts were applied to the column, and then the column was washed twice with wash buffer (50 mM sodium phosphate, 500 mM NaCl, 50 mM imidazole, 20% ethanol, pH 7.5). The His-tagged protein was eluted with elution buffer (50 mM sodium phosphate, 500 mM NaCl, 500 mM imidazole, pH 7.5).

### GAPDH enzyme assays

The activity of the glyceraldehyde-3-phosphate dehydrogenase enzyme (GAPDH EC 1.2.1.12) was measured at 55 °C as previously described [42]. The standard assay (200 µL working volume) contained 50 mM Tris–HCl pH 7.0, 10 mM sodium arsenate, 10 mM glyceraldehyde-3-phosphate, and 0.5 mM NAD^+^. To avoid thermal destruction of glyceraldehyde-3-phosphate, this substrate was added to the mixture immediately before starting the enzyme reaction. The formation NADH was followed by photometric observation at 340 nm (*ε* = 6.2 mM^−1^ cm^−1^) in a BioTek PowerWave XS plate reader (BioTek Instruments Inc., Winooski, VT, USA). The protein concentration was determined using the Bradford protein reagent with bovine serum albumin as the standard (Bio-Rad, Hercules, CA, USA). For the NADH inhibition test, 0 to 0.5 mM of NADH was added to the reaction mix. To avoid the saturating the detector, the wavelength was changed from 340 nm to 380 nm (*ε* = 1.2 mM^−1^ cm^−1^).

## Results

### Ethanol tolerance test

**Fig. 1:**
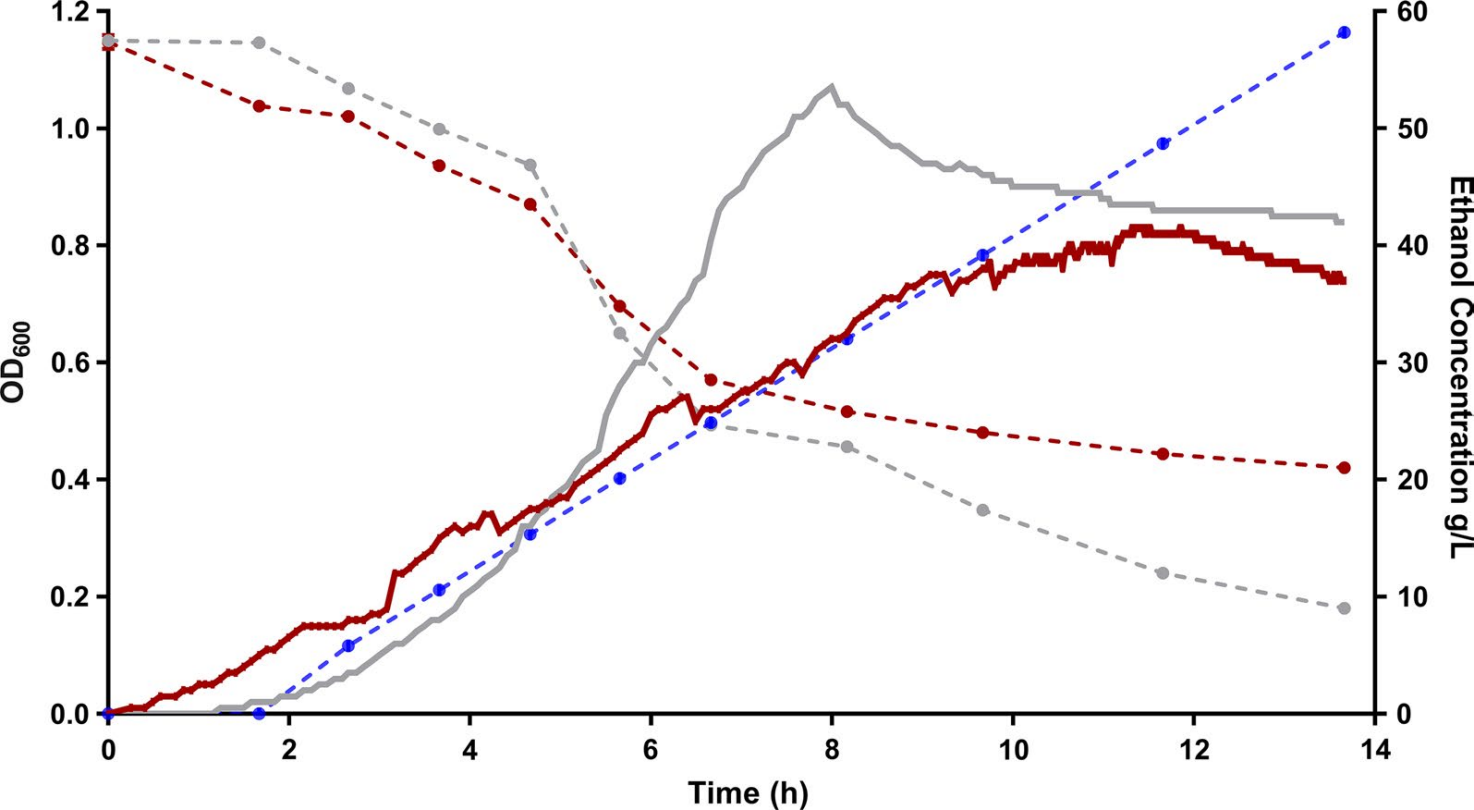
Growth test in LC medium with 10 g/L cellobiose. Ethanol was added to the culture at a rate of 5 g/L/h. The blue dashed line indicates the measured ethanol concentration in the culture with ethanol addition. The measured ethanol concentration in the culture without ethanol addition is not shown in the figure, since the final ethanol titer for that culture was only 1.2 g/L. The data presented here are a representative example of biological triplicates. Similar trends were found in all replicates

In order to characterize the metabolic response to ethanol, ethanol was added continuously to a growing culture at a rate of 5 g/L/h. This rate was chosen to achieve growth inhibition before the substrate carbon was completely consumed. As seen in Fig. 1, the presence of added ethanol reduced the maximum optical density achieved (OD_max_) and slowed growth. Increasing optical density was observed until the ethanol concentration reached 45 g/L.

### Metabolite analysis

**Fig. 2:**
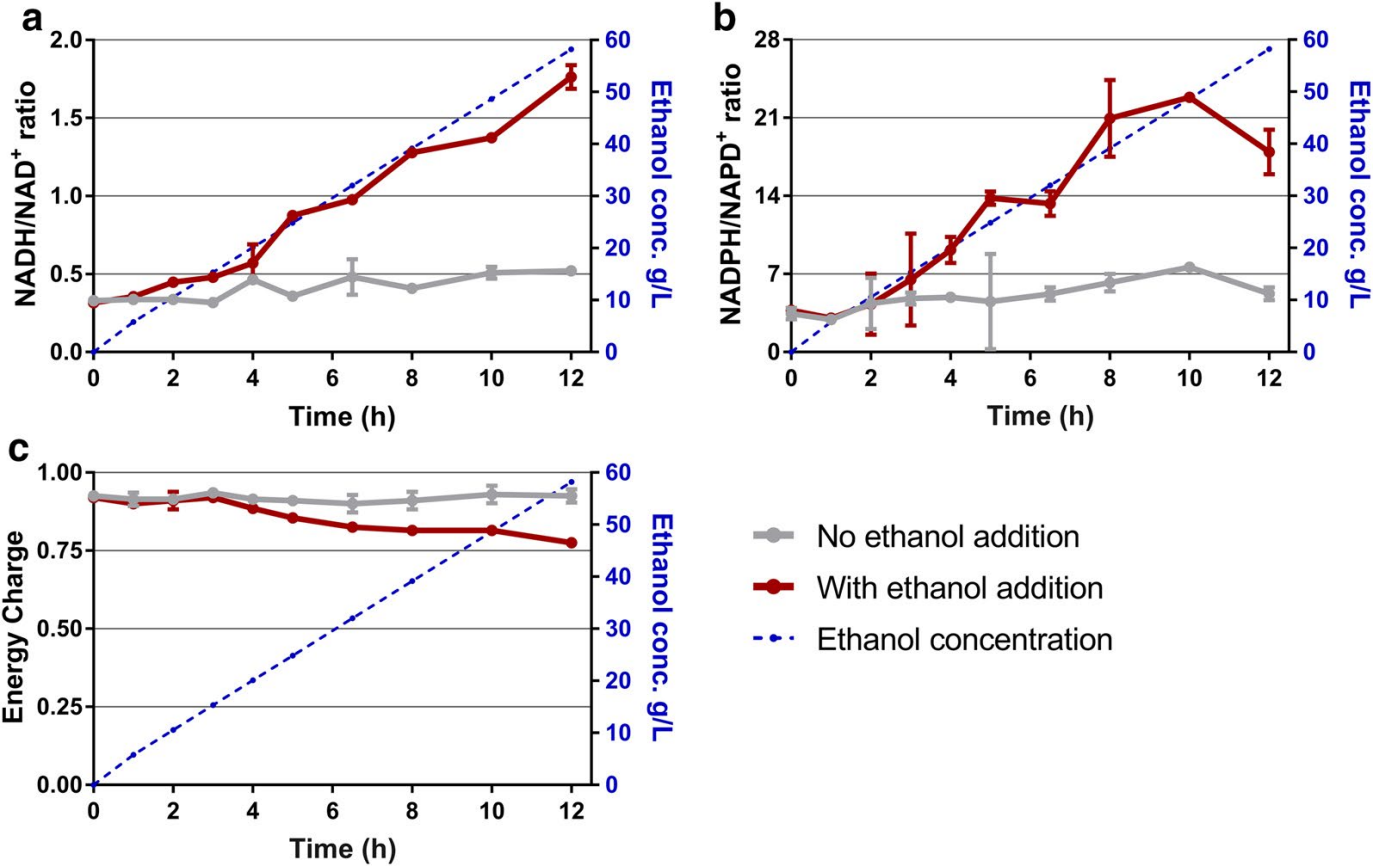
Nicotinamide cofactor ratios (**a**, **b**) and energy charges changes (**c**) in *C. thermocellum* in the presence of added ethanol. The blue dashed line indicates the measured ethanol concentration of the culture with ethanol addition. Error bars represent one standard deviation, *n* = 3 biological triplicates

Samples from the experiment depicted in Fig. 1 were collected at 1–2 h intervals, and intracellular metabolites were analyzed using LC–MS. The initial timepoint (T0) samples were taken after growth had started and immediately before ethanol addition. As ethanol was added, we observed a marked increase in the ratios of NADH/NAD^+^ and NADPH/NADP^+^ as well as a moderate decline in the energy charge compared to control strains not exposed to ethanol (Fig. 2a, b).

Both the NADH/NAD^+^ ratio and NADPH/NADP^+^ ratios increased significantly as the concentration of added ethanol increased (*t* test values of the endpoint samples were *p* = 0.02 and *p* = 0.0003, respectively) when the ethanol was added to the culture. The observed decrease in energy charge in response to increasing ethanol was highly significant (*p* value for the endpoint samples was 0.01) (Fig. 2c).

### Comparison of ethanol inhibition in *C. thermocellum* and *T. saccharolyticum*

To put the results from *C. thermocellum* in context, we performed the same ethanol inhibition experiment on an engineered strain of *T. saccharolyticum*, an organism that has been shown to be able to produce ethanol at up to 70 g/L more than 2.5 times greater than the maximum reported for *C. thermocellum* [15].

**Fig. 3:**
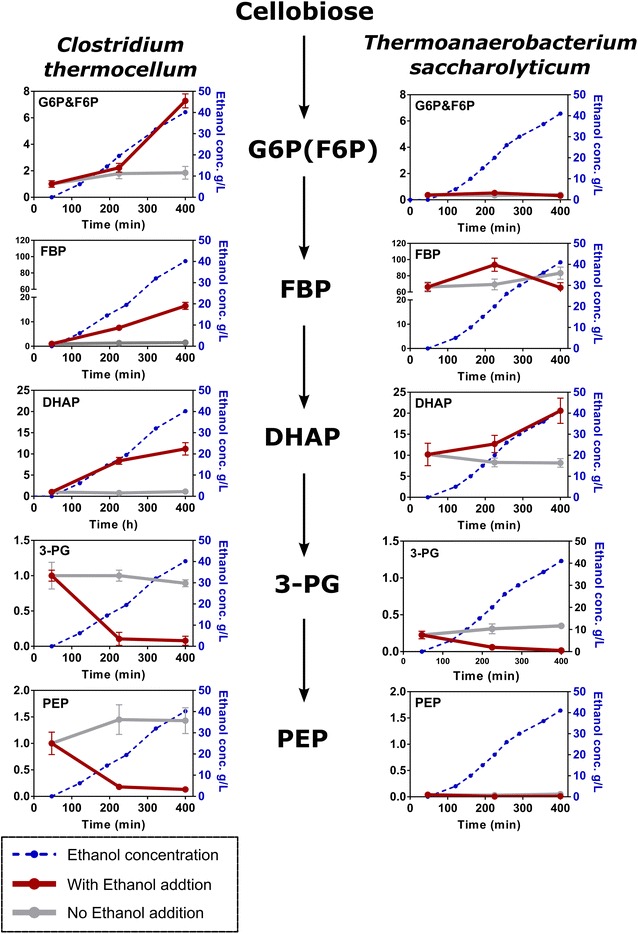
Relative concentrations of intracellular metabolites for *C. thermocellum* and *T. saccharolyticum* in the presence of increasing ethanol concentrations. For each metabolite, values were normalized to the *C. thermocellum* T0 samples. Thus, the vertical axis represents a (unitless) ratio of metabolite concentrations. Error bars represent one standard deviation, *n* = 3 biological triplicates. *G6P* glucose 6-phosphate, *F6P* fructose 6-phosphate, *FBP* fructose-1,6-bisphosphate, *DHAP* dihydroxyacetone phosphate, *G3P* glyceraldehyde 3-phosphate, *3-PG* 3-phosphoglycerate, *PEP* phosphoenolpyruvate

For both *C. thermocellum* and *T. saccharolyticum,* the relative concentration of 3-phosphoglycerate (3-PG) and phosphoenolpyruvate (PEP) decreased with increasing ethanol, indicative of a flux bottleneck upstream of 3-PG (Fig. 3). Marked differences were observed in the response of other metabolite concentrations. For *C. thermocellum,* the relative concentrations of the glucose-6-phosphate and fructose-6-phosphate (G6P/F6P) pool, the fructose-1,6-phosphate (FBP) pool, and the dihydroxyacetone phosphate (DHAP) pool increased by 7, 16, and 11-fold, respectively, between 0 and 400 min (0 and 40 g/L added ethanol). For *T. saccharolyticum* exposed to the same range of ethanol concentrations, no increases were found in the G6P/F6P and FBP pool while the DHAP pools increased by only twofold. Due to technical limitations of the LC–MS instrument, glyceraldehyde 3-phosphate (G3P) was not measured.

The different response of metabolite concentrations, to changes in added ethanol noted above, is consistent with a metabolic bottleneck in one of the steps in glycolysis between DHAP and 3-PG; that is, in reactions mediated by glyceraldehyde 3-phosphate dehydrogenase (GAPDH) or phosphoglycerate kinase (PGK).

Because the GAPDH reaction involves a nicotinamide cofactor and the redox state of such cofactors changed markedly in response to ethanol for both *C. thermocellum* and *T. saccharolyticum* (Additional file 4: Figure S1), we hypothesized that this enzyme (rather than PGK) might explain the difference in ethanol tolerance between the two strains.

### Comparison of Gapdh protein from *C. thermocellum* and *T. saccharolyticum*

**Fig. 4:**
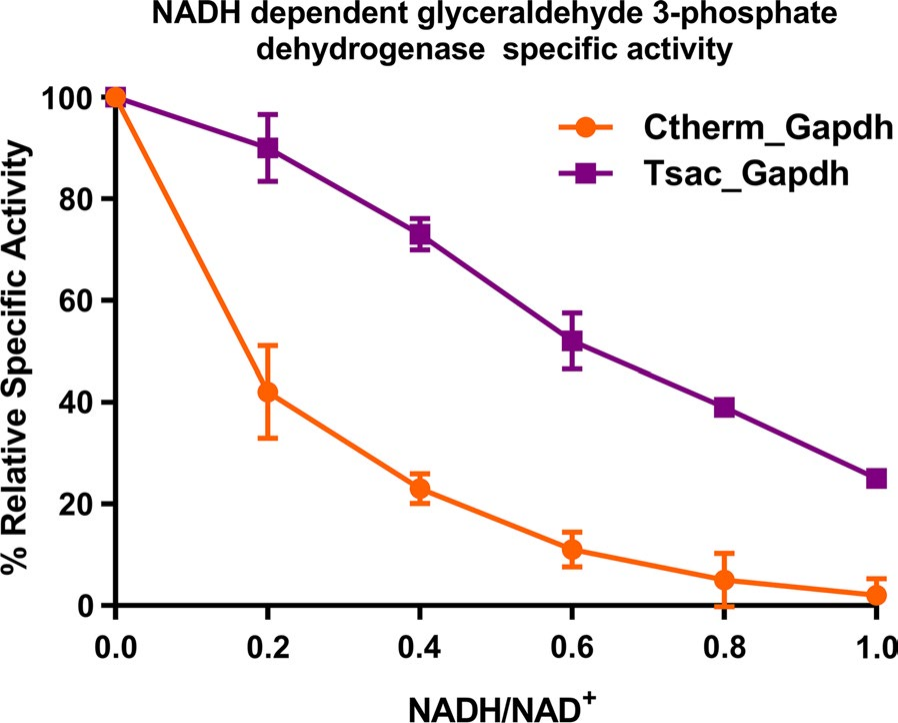
Comparison of glyceraldehyde 3-phosphate dehydrogenase specific activities under different NADH/NAD^+^ ratios. Error bars represent one standard deviation, *n* = 3 biological triplicates

The *gapdh* genes from *C. thermocellum* (Clo1313_2095) and *T. saccharolyticum* (Tsac_2486) were expressed and purified in *E. coli*. The values of *K*_m_ and *V*_max_ for *C. thermocellum* Gapdh were 0.6 ± 0.1 mM and 17.8 ± 2.1 S^−1^, respectively. The Gapdh of *T. saccharolyticum* exhibited similar kinetics with a *K*_m_ of 1.4 ± 0.3 mM and a *V*_max_ of 8.0 ± 1.3 S^−1^. To compare their response to inhibition, their specific activities were measured with different NADH/NAD^+^ ratios (Fig. 4).

As may be seen from Fig. 4, the Gapdh from *C. thermocellum* (Ctherm_Gapdh) was much more sensitive to the NADH/NAD^+^ ratio. More than half of the activity was lost when the ratio was 0.2, whereas the Gapdh from *T. saccharolyticum* (Tsac_Gapdh) still had more than 90% of activity remaining. At a ratio of 1.0, the Ctherm_Gapdh lost all activity, while 30% of activity remained for the Tsac_Gapdh.

**Fig. 5:**
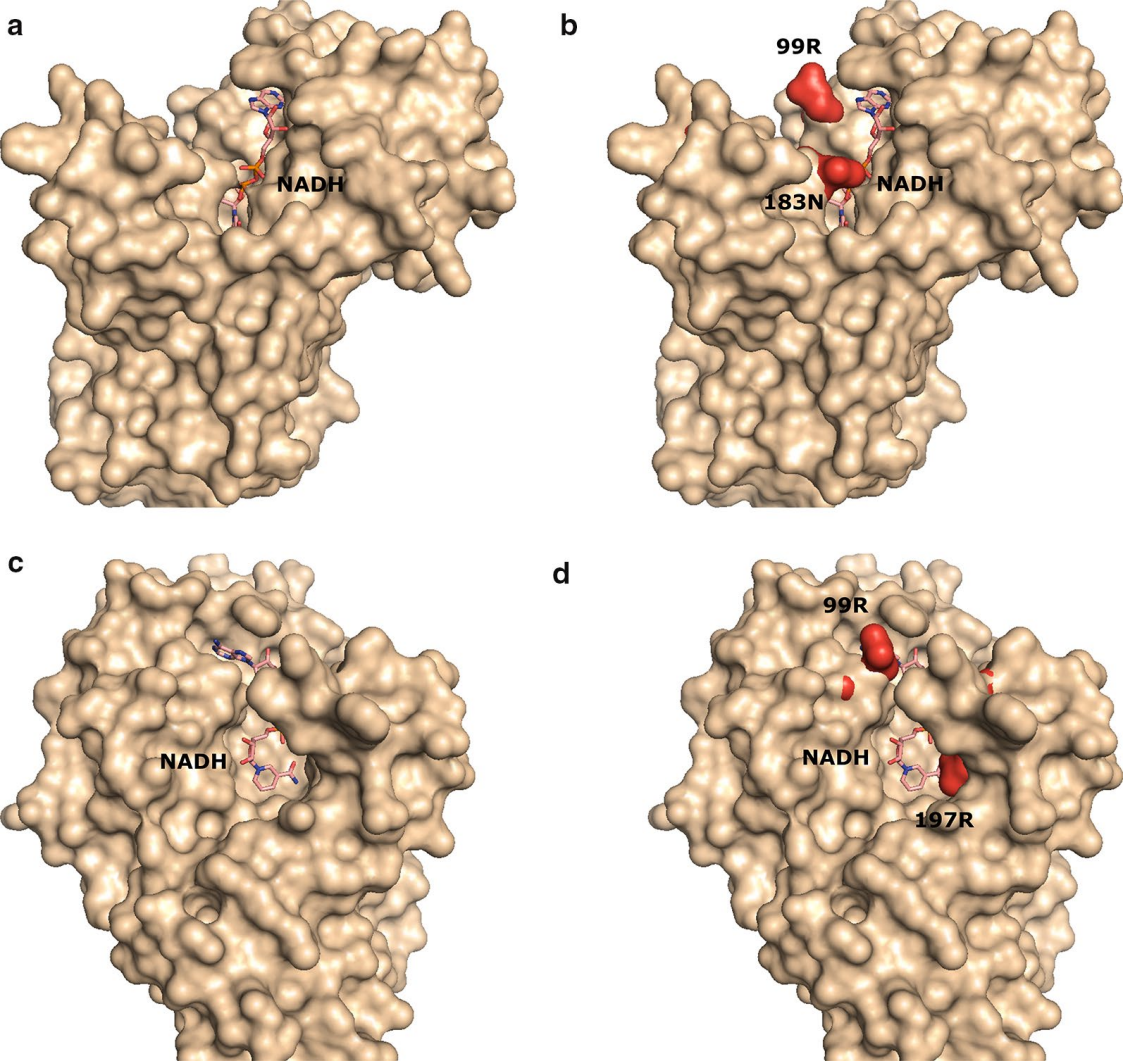
Homology modeling comparison of Gapdh from *C. thermocellum* and *T. saccharolyticum.* **a**, **c** Structures of Ctherm_Gapdh from two different angles. **b**, **d** Structures of Tsac_Gapdh overlaid on Ctherm_Gapdh. Key differences are shown in red

To analyze differences in the structure of the Gapdh proteins, homology models of the Ctherm_Gapdh and Tsac_Gapdh were constructed using the crystal structures of *Oryza Sativa* (Protein Data Bank code 3E5R, sharing 72.89% of identity) and *Bacillus stearothermophilus* (Protein Data Bank code 1GD1, sharing 79.52% of identity), respectively [43]. These two models were aligned for comparison. Figure 5a, c illustrates the structure of Ctherm_Gapdh from different angles. The location of the NADH cofactor in the active site can be clearly seen. Tsac_Gapdh had a similar catalytic cavity for NADH but with several differences in residues 99R, 183N, and 197R (Fig. 5b, d, indicated in red). These three residues are much larger than the corresponding residues in Ctherm_Gapdh (99V, 181A, and 196G) and these substitutions may partially restrict the entrance channel for NADH which may explain the higher *K*_m_. However, this change may also be the reason why Tsac_Gapdh was less sensitive to inhibition by high NADH/NAD^+^ ratios [44].

### The performance of *T. saccharolyticum* Gapdh in *C. thermocellum*

**Fig. 6:**
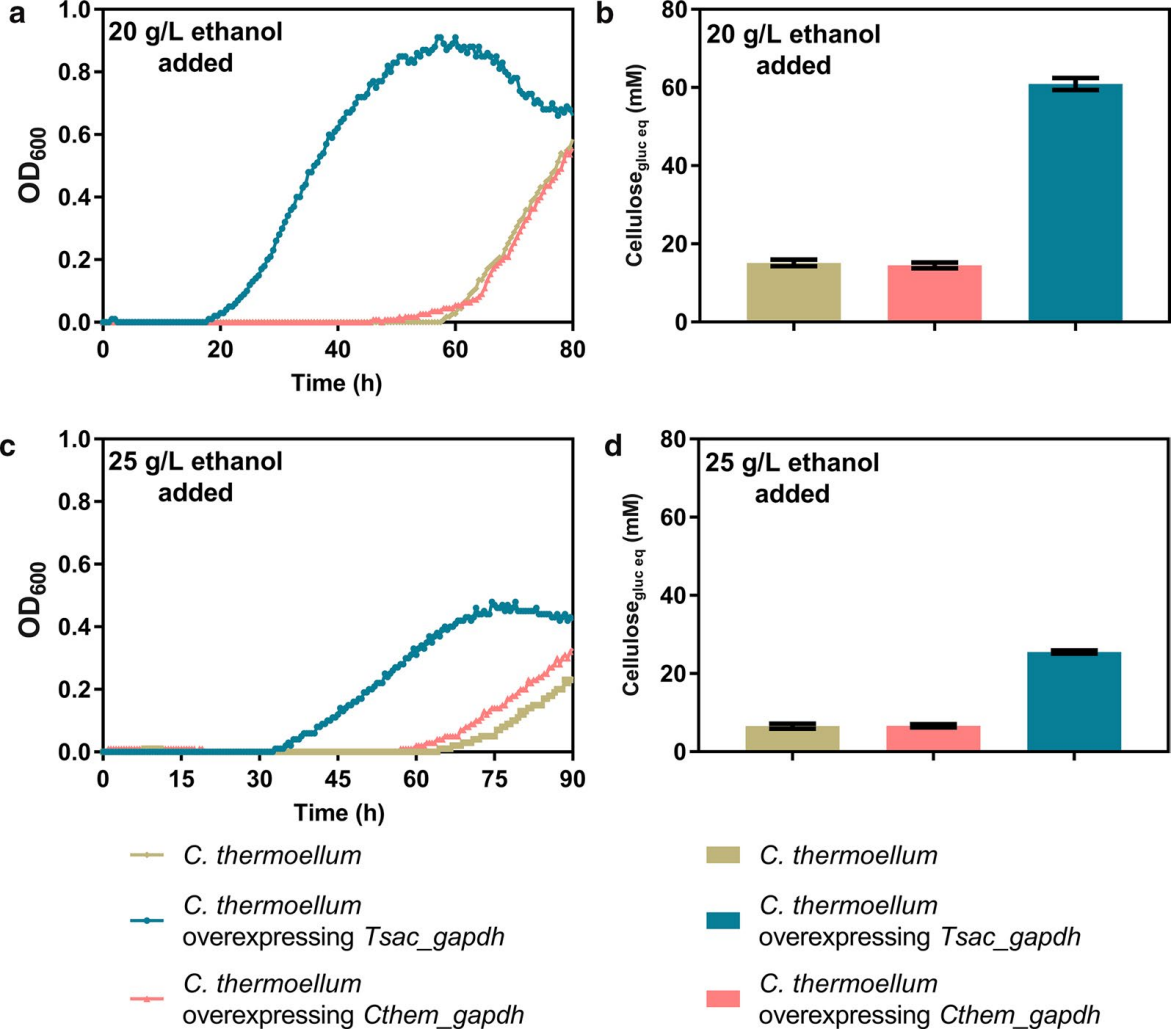
Growth of *C. thermocellum* strains expressing different *gapdh* genes in the presence of added ethanol. The data presented in panels **a**, **c** are representative examples of growth curve data. Similar trends were found in all replicates. The data presented in panels **b**, **d** are averages of cellulose consumption after 80 h. Error bars represent one standard deviation, *n* = 3 biological triplicates

The *T. saccharolyticum gapdh* was expressed in *C. thermocellum* to see if it would improve ethanol tolerance. Wild-type *C. thermocellum* and a strain overexpressing native *gapdh* were used as controls. The resulting strains were assayed for their ability to grow in the presence of 20 or 25 g/L added ethanol (Fig. 6). The strain carrying the *T. saccharolyticum gapdh* showed improvement in growth at both concentrations of added ethanol. In addition, this strain consumed more cellulose after 80 h culture compared to the control strains.

To determine whether the increase in ethanol tolerance would affect ethanol production, the engineered strain and the wild-type control strain were both cultured in 50 g/L Avicel in a bioreactor. Compared to the control strain, expression of the *T. saccharolyticum gapdh* increased ethanol titer by 28% (from 7.7 ± 0.3 to 9.9 ± 0.5 g/L) (Additional file 5: Figure S2).

## Discussion and conclusions

In this work, we analyzed the effect of added ethanol on the distribution of intracellular metabolites in *C. thermocellum*. As reviewed in the introduction, wild-type cultures of *C. thermocellum* and other thermophilic bacteria are generally able to initiate growth in the presence of only about 20 g/L ethanol, but readily develop the ability to initiate growth at ethanol concentrations at least twofold higher after serial transfer over a period of weeks. We [18, 21] and others [19] have attributed this to genetic mutation followed by selection, and the mechanism has been confirmed by identifying a point mutation that confers the ethanol-tolerant phenotype in the absence of selection [21]. However, we observed in this study the ability to grow at ethanol concentrations exceeding 20 g/L after exposure to ethanol for only 4 h, which is very likely too short to be explained by mutation and selection [45]. Identifying the tolerance mechanism operative in the experiments described herein, as well as why tolerance is substantially greater to continuously added ethanol than to ethanol present initially, are interesting questions for future work, with important applied implications.

One of the leading hypotheses for ethanol inhibition is disruption of the cell membrane, which leads to loss of the proton motive force and subsequent decreased ability to generate ATP [27]. By directly measuring the adenylate charge, we have shown that added ethanol has no effect on ATP at concentrations below 15 g/L and a negligible effect at higher concentrations (Fig. 2), which suggests that membrane disruption is not the primary cause of ethanol inhibition in *C. thermocellum*, at least for ethanol concentrations up to 45 g/L.

Another hypothesis for the mechanism of ethanol inhibition is that it affects specific metabolic enzymes. We observed a dramatic accumulation of NADH and NADPH when ethanol was added to the culture. Ethanol production in *C. thermocellum* involves successive reduction of acetyl-CoA and acetaldehyde with electrons provided by NADH (i.e., the ALDH and ADH reactions). The observed increase in the NADH/NAD^+^ ratio in response to increasing ethanol is consistent with mass action. The concomitant increase in NADPH/NADP^+^ ratios is interesting to note and suggests that electrons may be exchanged between the two nicotinamide cofactor pools and/or that the oxidation state of these pools is controlled by a common factor.

Since NADH is known to be a competitive inhibitor of the GAPDH reaction [46–48], and we observe accumulation of metabolites upstream of the GAPDH reaction and depletion of reactions downstream of the GAPDH reaction, we conclude that, in the range of concentrations tested, ethanol inhibits *C. thermocellum* metabolism at the GAPDH reaction. Furthermore, *T. saccharolyticum* is more resistant to ethanol inhibition than *C. thermocellum* and also has a Gapdh enzyme that is more resistant to inhibition by high levels of NADH. To confirm this hypothesis, the *T. saccharolyticum gapdh* gene was overexpressed in *C. thermocellum,* resulting in dramatic improvements in ethanol tolerance: growth was initiated sooner, the growth rate was faster and substrate consumption was fourfold higher (Fig. 6).

Finally, we showed that this insight can be exploited for the practical purpose of increasing ethanol titer in *C. thermocellum*. Expressing the *T. saccharolyticum gapdh* gene in *C. thermocellum* increased ethanol titer by 28%. Although this result by itself is a useful strategy for metabolic engineering of *C. thermocellum*, it also suggests a future line of work where engineering the Gapdh enzyme for improved tolerance to NADH could be used to further increase ethanol production *C. thermocellum*.

## Additional files

**Additional file 1: Table S1.** Strain used in this work.

**Additional file 2: Table S2.** Plasmids used in this study.

**Additional file 3: Table S3.** Primers used in this study.

**Additional file 4: Figure S1.** Comparison of nicotinamide cofactor ratios between *C. thermocellum* and *T. saccharolyticum* in the presence of added ethanol. The absolute concentration of nicotinamide cofactors was determined based on a standard curve and normalized to cell number as determined by qPCR. Error bars represent one standard deviation, *n* = 3 biological triplicates.

**Additional file 5: Figure S2.** Fermentation profiles of *C. thermocellum* with or without the *tsac_gapdh* gene. Cells were grown in batch pH-controlled fermenters with 50 g/L Avicel. Error bars represent one standard deviation, *n* = 3 biological triplicates.

## Abbreviations

ADH: alcohol dehydrogenase
ALDH: aldehyde dehydrogenase
BSA: bovine serum albumin
CBP: consolidated bioprocessing
GAPDH: glyceraldehyde 3-phosphate dehydrogenase
PGK: phosphoglycerate kinase
MOPS: 3-morpholino-propane-1-sulfonic acid
G6P: glucose 6-phosphate
F6P: fructose 6-phosphate
FBP: fructose-1,6-bisphosphate
DHAP: dihydroxyacetone phosphate
G3P: glyceraldehyde 3-phosphate
3-PG: 3-phosphoglycerate
PEP: phosphoenolpyruvic acid
