# Supplementary figures and images for: Metabolome analysis reveals a role for glyceraldehyde 3-phosphate dehydrogenase in the inhibition of C. thermocellum by ethanol
Source: Biotechnol Biofuels. 2017 Nov 30;10:276. doi: 10.1186/s13068-017-0961-3 (PMC5708176; doi:10.1186/s13068-017-0961-3)

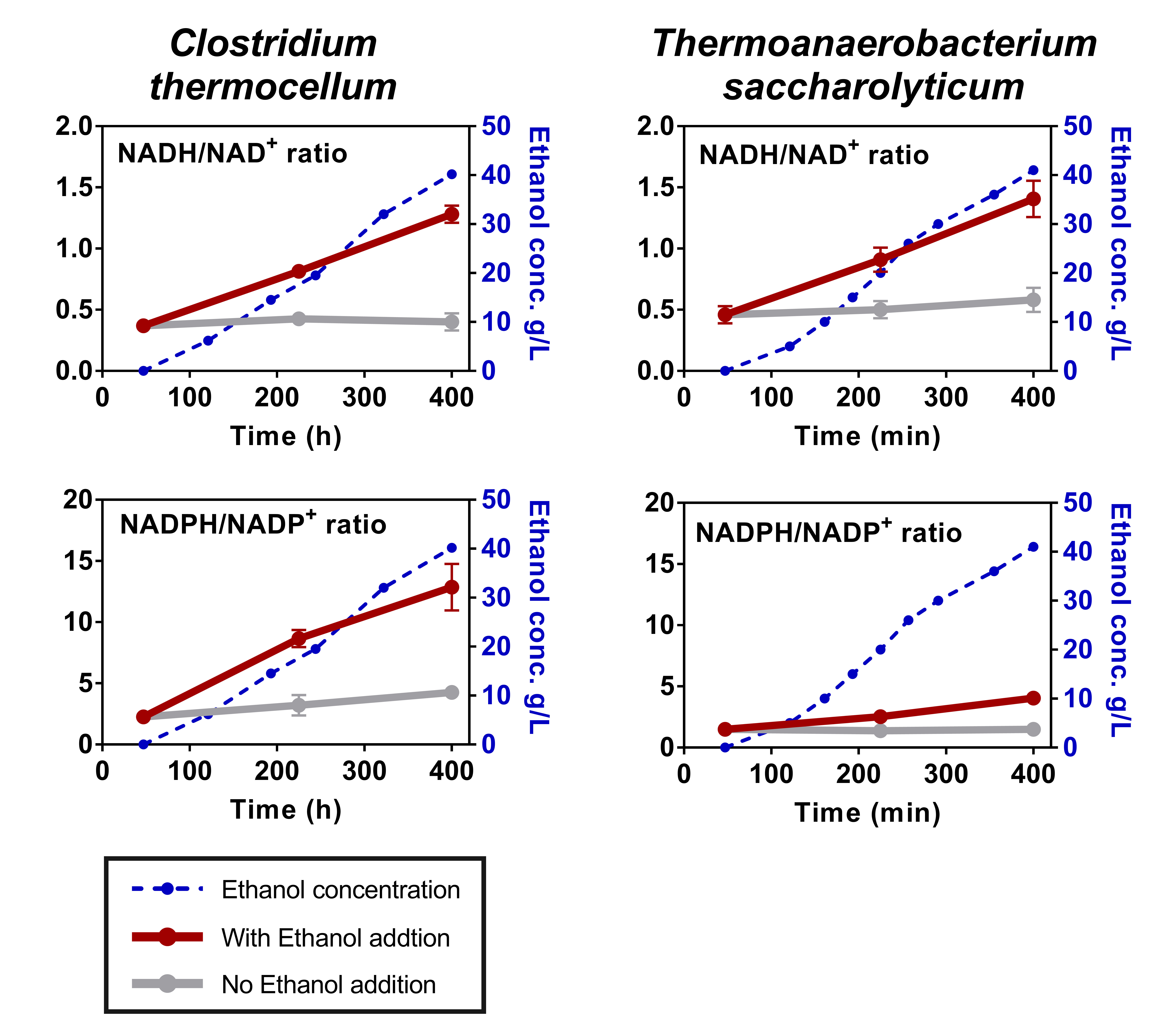

Supplement: Supplementary file 4 — Additional file 4: Figure S1. Comparison of nicotinamide cofactor ratios between C. thermocellum and T. saccharolyticum in the presence of added ethanol. The absolute concentration of nicotinamide cofactors was determined based on a standard curve and normalized to cell number as determined by qPCR. Error bars represent one standard deviation, n = 3 biological triplicates. [file 13068_2017_961_MOESM4_ESM.tif]

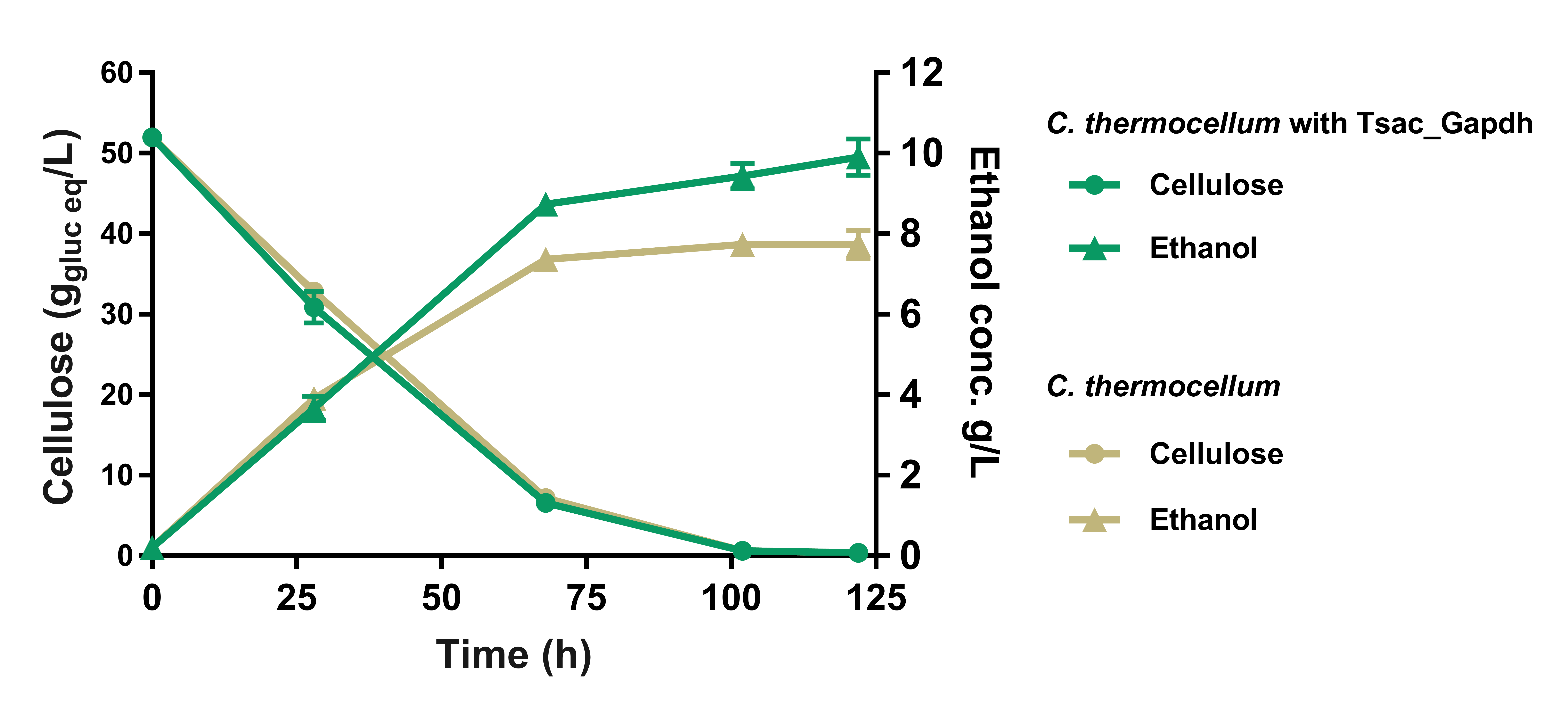

Supplement: Supplementary file 5 — Additional file 5: Figure S2. Fermentation profiles of C. thermocellum with or without the tsac_gapdh gene. Cells were grown in batch pH-controlled fermenters with 50 g/L Avicel. Error bars represent one standard deviation, n = 3 biological triplicates. [file 13068_2017_961_MOESM5_ESM.tif]
